# Profile of Medicaid enrollees with sickle cell disease: A high need, high cost population

**DOI:** 10.1371/journal.pone.0257796

**Published:** 2021-10-27

**Authors:** April Grady, Anthony Fiori, Dhaval Patel, Jessica Nysenbaum

**Affiliations:** 1 Manatt Health Strategies, Washington, DC, United States of America; 2 Manatt Health Strategies, New York, New York, United States of America; University of Tennessee Health Science Center, UNITED STATES

## Abstract

Sickle cell disease is a progressively debilitating genetic condition that affects red blood cells and can result in a variety of serious medical complications, reduced life expectancy, and diminished quality of life. Medicaid nationwide covered 66 percent of sickle cell disease hospitalizations in 2004 and 58 percent of emergency department visits for the disease between 1999 and 2007. Using Medicaid data from four states with large populations that account for more than one-third of Medicaid program enrollment, we examined the characteristics of those with sickle cell disease. We found instances of mortality rates more than nine times the age-adjusted population average (in Texas, a mortality rate for Medicaid enrollees with SCD of 1.11 percent compared to 0.12 percent overall); rates of disability-related eligibility–which is associated with long-term Medicaid enrollment–of up to 69 percent; and half or more of affected enrollees having (all-cause) hospital stays, emergency department visits, and opioid prescription fills. With gene therapies on the horizon that will spur discussions of treatment coverage, costs, and outcomes for people with sickle cell disease, it is important for relevant stakeholders to understand the affected populations.

## Introduction

Sickle cell disease (SCD) is a genetic disorder that affects the structure and life span of red blood cells, thereby impairing blood flow and causing other issues such as blood vessel damage. This results in episodes of severe pain and a variety of potentially life-threatening health problems that include acute chest syndrome (a form of lung injury) and stroke, as well as kidney disease and other chronic complications that lead to progressive organ damage [1]. SCD most commonly affects individuals whose ancestors came from sub-Saharan Africa and certain other areas of the world where malaria is or was common. Approximately 100,000 individuals in the United States are estimated to have SCD, with potentially more than 90 percent being African American [2].

In the United States, although mortality rates for children with SCD have declined in recent decades, those for adults with SCD have increased, and half of people with SCD do not live past the age of 40 [3]. Deficiencies in quality of care for the population with SCD are well-documented and include poor access to knowledgeable health care providers, inadequate treatment of the disease’s effects on the body and of associated pain, and discrimination [4, 5]. Quality of life is also a concern in light of the negative effects that SCD has on education, employment, and relationships [6]. Up to 40 percent of people with SCD meet the medical condition criteria for publicly funded disability benefits that are available under certain circumstances for children with functional impairments and adults with a limited ability to work [7]. SCD also affects the family members of people with the condition–particularly parents of children with SCD–who may face financial and other difficulties as a result of their caregiving responsibilities [8].

Although SCD is the most common inherited blood disorder in the United States, there are substantial gaps in knowledge about the people who are affected by the disease–for example, where they use services, whether they receive recommended treatments, and how their co-morbid conditions and health outcomes vary. Medicaid plays a significant role in funding treatment for people with SCD, paying for 66 percent of all SCD hospitalizations in 2004 and 58 percent of SCD emergency department visits between 1999 and 2007 [9, 10]. Medicaid is a joint federal and state program that provides health insurance to low-income individuals in qualifying groups such as pregnant women, children, parents, and individuals with disabilities; states can also cover childless adults who do not fall into these groups. Eligibility is typically reassessed at least once per year and may occur sooner if an enrollee has an increase in income or other change in circumstance. Some individuals enrolled in Medicaid are also enrolled in Medicare, a federal program providing health insurance to individuals over 65 and individuals with disabilities. Individuals enrolled in both programs are known as dual eligibles, as they qualify for both Medicaid and Medicare.

We aim here to address some gaps in knowledge about the SCD population with a profile of Medicaid enrollees with SCD in four states selected in part for their large populations that account for more than one-third of all people enrolled in Medicaid nationwide [11, 12].

## Data and methods

This analysis uses the Medicaid Analytic eXtract (MAX). MAX is a research ready data source developed by the federal Centers for Medicare & Medicaid Services (CMS) that contains enrollment and claims data for all individuals enrolled in Medicaid in a given calendar year [12]. The study was determined exempt from Institutional Review Board (IRB) oversight by the Advarra IRB, which granted a waiver of Health Insurance Portability and Accountability Act (HIPAA) authorization, and it was conducted under a CMS data use agreement (DUA) applicable to Research Identifiable Files (RIFs). In accordance with the DUA, cell sizes of less than 11 were suppressed in the analytic output to protect beneficiary confidentiality.

Data from the most recent year available at the time of the analysis was examined for each state (California– 2014, Florida– 2012, New York– 2013, Texas– 2012). Data for prior years, reaching back to 2011, was also examined (e.g., CA data was analyzed for 2011–2014, NY data was analyzed for 2011–2013). MAX includes both fee-for-service (FFS) claims that represent services paid directly by state Medicaid agencies and encounter records that represent services paid by Medicaid managed care plans on behalf of states. Due to a history of incomplete encounter data in the MAX files, we assessed each state’s managed care encounter data and confirmed that it met specified completeness and quality thresholds for analytic purposes [13]. Checks of the encounter data included comparisons of aggregate record counts, average number of records per enrollee, and the share of records with diagnosis and procedure codes against specified benchmarks. The methods used mirrored those of researchers who have conducted research for CMS and the Medicaid and CHIP Payment and Access Commission. In MAX, payments by states to managed care plans under capitated arrangements are available, but payments by managed care plans to providers are not. Because capitated payments generally reflect population averages for broad groups of managed care plan enrollees (e.g., children, non-disabled adults, disabled adults) rather than disease-specific or other subgroups, they are likely to understate underlying medical costs of the SCD population.

Using the MAX data for California, Florida, New York, and Texas, we identified the SCD enrollee population in each state using a combination of diagnosis and procedure codes. ICD-9 diagnosis codes of 282.60, 282.61, 282.62, 282.63, 282.64, 282.68, 282.69, 282.41, and 282.42 were used to identify a sickle cell disease diagnosis. The presence of a sickle cell disease diagnosis was required on at least two fee-for-service claims or managed care encounter records on different dates, to exclude potential “false positive” cases associated with diagnostic testing. Using two or more claims is a common method, as utilized by CMS in a September 2020 infographic with statistics on the Medicaid SCD population [14]. An exception was made for individuals whose only SCD claims were on the same date but who had at least one SCD claim for inpatient care, one drug claim for hydroxyurea based on an applicable national drug code (NDC), or six claims (any diagnosis) for transfusion care based on an applicable procedure code.

After determining the prevalence of individuals with SCD, a series of statistics described below were run to determine the eligibility and enrollment characteristics of the SCD population compared to all Medicaid enrollees. The distribution of enrollees’ eligibility pathway to qualify for Medicaid was calculated using the basis of eligibility code variable. Length of time across years enrolled in Medicaid was assessed by taking individuals enrolled in the latest time period in a given state and checking the proportion present in prior year data files. This step used the unique encrypted beneficiary identifier provided in the data to link files across years. Statistics were also calculated on the proportion of SCD enrollees dually eligible for both Medicaid and Medicare using the dual eligibility variable, and the proportion of SCD enrollees with full versus partial Medicaid benefits using the restricted benefits flag. SCD enrollees’ managed care enrollment status was determined based on the managed care combinations flag. Mortality rates for the SCD Medicaid enrollee population were calculated as the number with a death indicator divided by the total; although this indicator is based on administrative data known to undercount deaths, it reflects the only available information in the MAX data source. Age-adjusted mortality rates for the overall population are state averages weighted to reflect the age distribution of the Medicaid SCD enrollee population [15].

Selected utilization metrics for SCD enrollees were calculated, including number of inpatient stays, visits to the emergency department (ED), use of outpatient drugs, and use of opioids. Inpatient stays were calculated based on the presence of any claims or encounters in the MAX inpatient (IP) file. Use of outpatient drugs were identified based on having claims or encounters in the prescription drug (RX) file. Fills for opioids were identified by analyzing RX claims with an NDC that mapped to the opioid drug class in MediSpan. The MediSpan database is a tool that facilitates mapping of NDC codes to drug names and drug classes [16]. Opioids that are used to treat opioid use disorder (belbuca, bunavail, buprenorphine, suboxone, and zubsolv) were excluded from the analysis of opioid fills. ED visits were identified based on having procedure codes between 99281–99285; having a revenue code of 045X or 0981; or having a procedure code between 10040–69979 in combination with a place of service code of 23.

As noted above, capitated payments available in the MAX data are likely to understate underlying medical costs of the SCD population enrolled in managed care plans. Results presented here are limited to expenditure amounts as reported in MAX (i.e., payments by states to providers under fee-for-service and to managed care plans under capitated arrangements). Payments by states to providers and to managed care plans were captured using the amount paid variable on FFS claims, which were identified using the type of claim variable.

## Results

The number of Medicaid enrollees with SCD across the four states analyzed is 14,369. Of Medicaid SCD enrollees in the states examined, dually eligible individuals account for between 6 percent (Texas) and 15 percent (California) of the total (Table 1). Because Medicare is the primary payer of drug and acute care costs for these individuals, Medicaid spending and utilization figures reported here for SCD enrollees exclude dual eligibles. They also exclude those who had only limited emergency or family planning benefits through Medicaid, or had outlier spending (reflecting one enrollee in New York).

**Table 1:** Eligibility and benefit characteristics of Medicaid enrollees with SCD.

Characteristic	California		Florida		New York		Texas	
	2014		2012		2013		2012	
	Number	%	Number	%	Number	%	Number	%
**Ever enrolled**								
Total	2,535	100.0%	4,165	100.0%	5,138	100.0%	2,531	100.0%
**Medicaid eligibility group**								
Age 65+	16	0.6%	16	0.4%	75	1.5%	--^a^	--^a^
Disabled, under age 65	1,498	59.1%	2,732	65.6%	2,524	49.1%	1,746	69.0%
Age 19–64	1,030	40.6%	1,460	35.1%	1,639	31.9%	946	37.4%
Under age 19	468	18.5%	1,272	30.5%	885	17.2%	800	31.6%
Other adult, 19–64	419	16.5%	312	7.5%	1,111	21.6%	119^a^	4.7%^a^
Other child, under 19	602	23.7%	1,105	26.5%	1,428	27.8%	666	26.3%
**Dual Medicaid-Medicare status and Medicaid benefit package**								
Dually eligible for Medicare	369	14.6%	473	11.4%	648	12.6%	157	6.2%
Not dually eligible for Medicare	2,166	85.4%	3,692	88.6%	4,490	87.4%	2,374	93.8%
Full Medicaid benefits^b^	2,141	84.5%	3,625	87.0%	4,392	85.5%	2,344	92.6%
Limited Medicaid benefits^c^	25	1.0%	67	1.6%	98	1.9%	30	1.2%
**Fee-for-service and managed care status** ^d^								
FFS only during year	501	19.8%	1,287	30.9%	845	16.4%	95	3.8%
FFS and limited managed care only	14	0.6%	1,603	38.5%	90	1.8%	764	30.2%
Comprehensive managed care	2,020	79.7%	1,275	30.6%	4,203	81.8%	1,672	66.1%

SCD is sickle cell disease. FFS is fee-for-service. Reflects the most recent month of Medicaid coverage unless noted otherwise.

^a^ Due to an enrollee count of less than 11 that must be suppressed, individuals age 65+ are included in the “Other adult, 19–64” group.

^b^ As with other Table 1 statistics, reflects most recent month of enrollment. Other figures reflect individuals who were non-dual with full benefits in all enrollment months, which is a slightly smaller number.

^c^ Primarily reflects individuals eligible only for emergency services due to immigration status, and a smaller number eligible only for family planning services.

^d^ Examples of limited managed care include standalone dental, behavioral health, and long-term care plans. Comprehensive managed care reflects one or more months of enrollment in a plan that covers a wider range of services, including inpatient hospital care.

Eligibility based on a disability is the most common Medicaid eligibility pathway for SCD enrollees in each state, ranging from 49 percent in New York to 69 percent in Texas.

**Fig 1:**
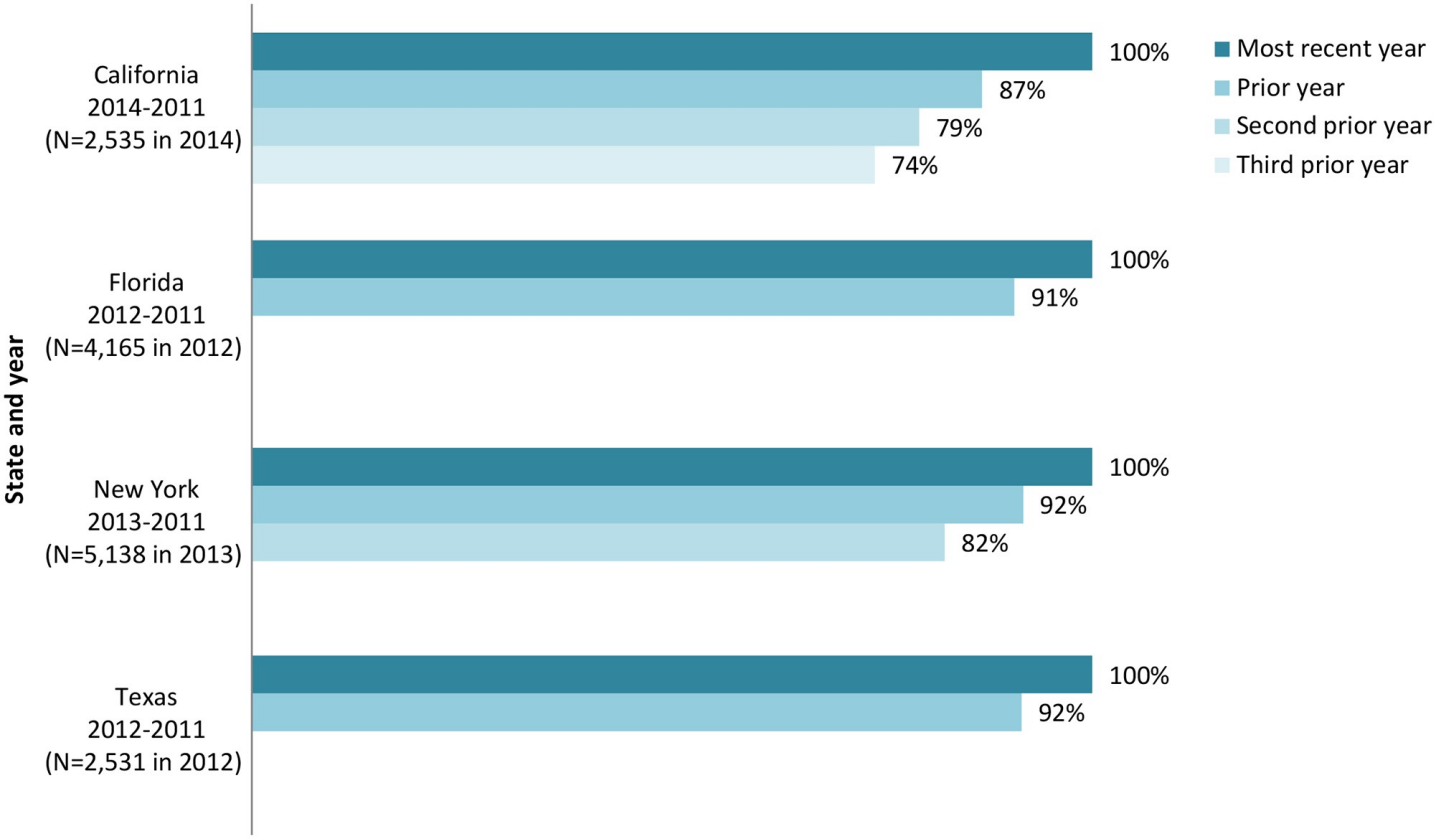
Among Medicaid enrollees with SCD, share with prior year(s) of enrollment. SCD is sickle cell disease.

For the available years of data, the share of SCD enrollees with at least one month of managed care enrollment ranges from 31 percent in Florida to 82 percent in New York (Table 1). Individuals with SCD tend to maintain their Medicaid coverage over time. Among those enrolled in a given year, the analysis shows that about 90 percent (87 percent in California, 91 percent in Florida, and 92 percent in both New York and Texas) were enrolled the prior year. Looking further back in time for California, for which longitudinal data were available, 74 percent of enrollees with SCD identified in 2014 were enrolled three years prior (i.e., in 2011) (Fig 1).

**Fig 2:**
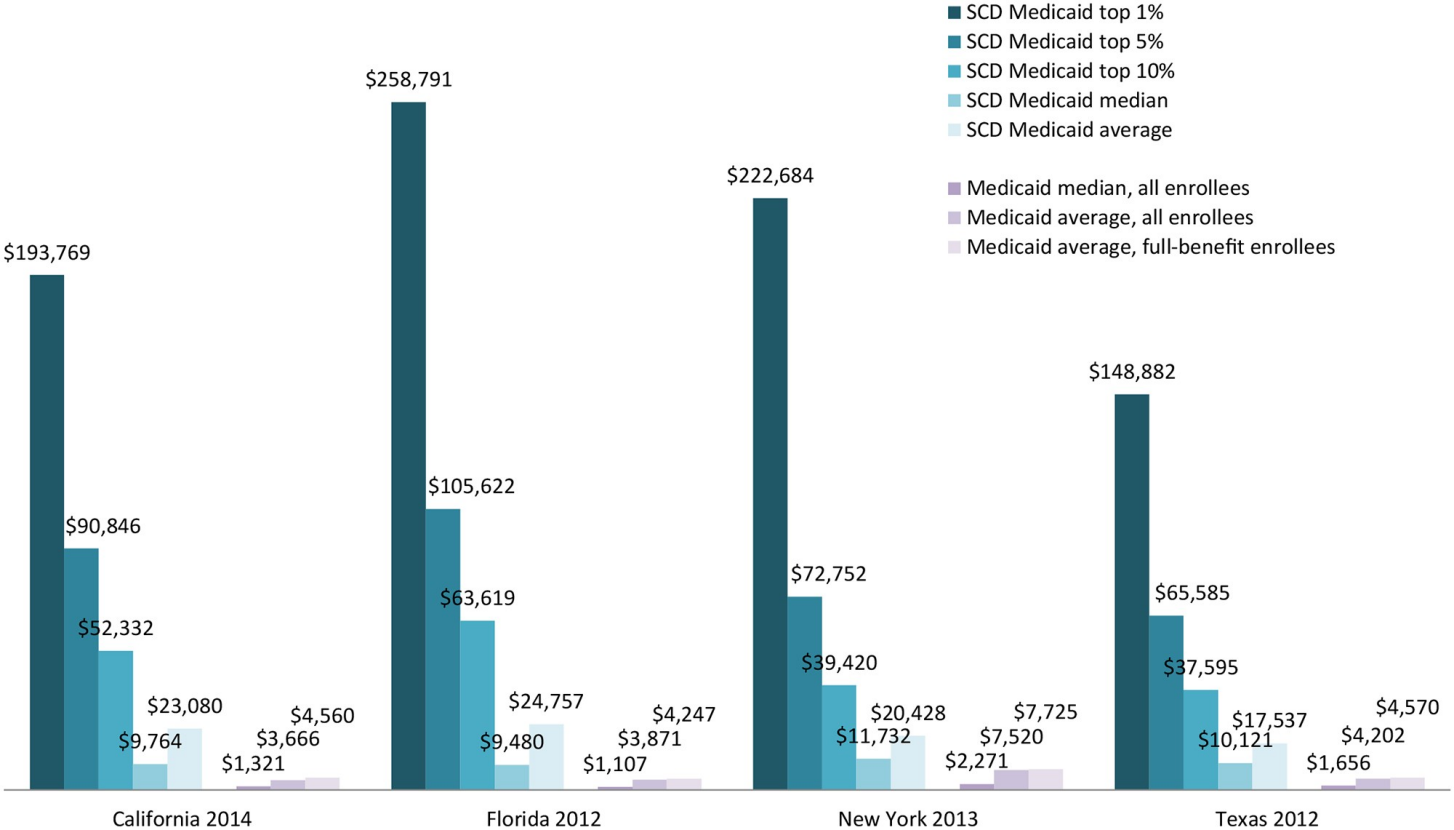
Annual Medicaid spending per enrollee, SCD relative to overall. SCD is sickle cell disease. All figures reflect payments by states to providers under fee-for-service arrangements and to plans under capitated managed care arrangements. As previously mentioned, capitated payments are likely to understate underlying medical costs of the SCD population.

**Fig 3:**
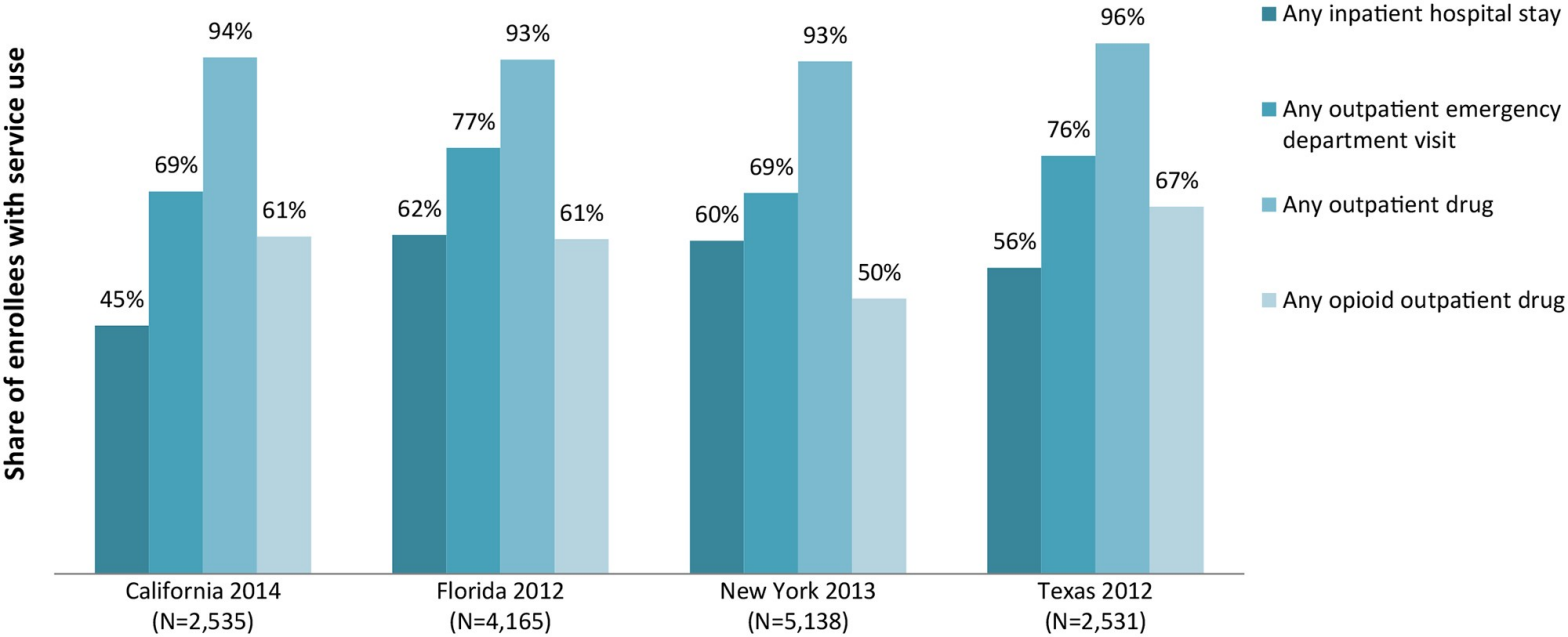
Selected Medicaid utilization metrics for non-dual, full-benefit Medicaid enrollees with SCD. SCD is sickle cell disease.

Medicaid enrollees with SCD in the four states examined have substantial utilization of hospital-based care, including inpatient stays (ranging from 45 percent to 62 percent) and outpatient emergency department visits (69 to 76 percent). They also have high utilization of prescription opioids (ranging from 50 percent to 67 percent) and other outpatient drugs (93 to 96 percent) (Fig 2). The population is also high-cost, as average Medicaid spending for enrollees with SCD can be more than five times the average for all Medicaid enrollees in a state (e.g., $24,800 versus $4,200 in Florida) (Figs 2 and 3).

**Fig 4:**
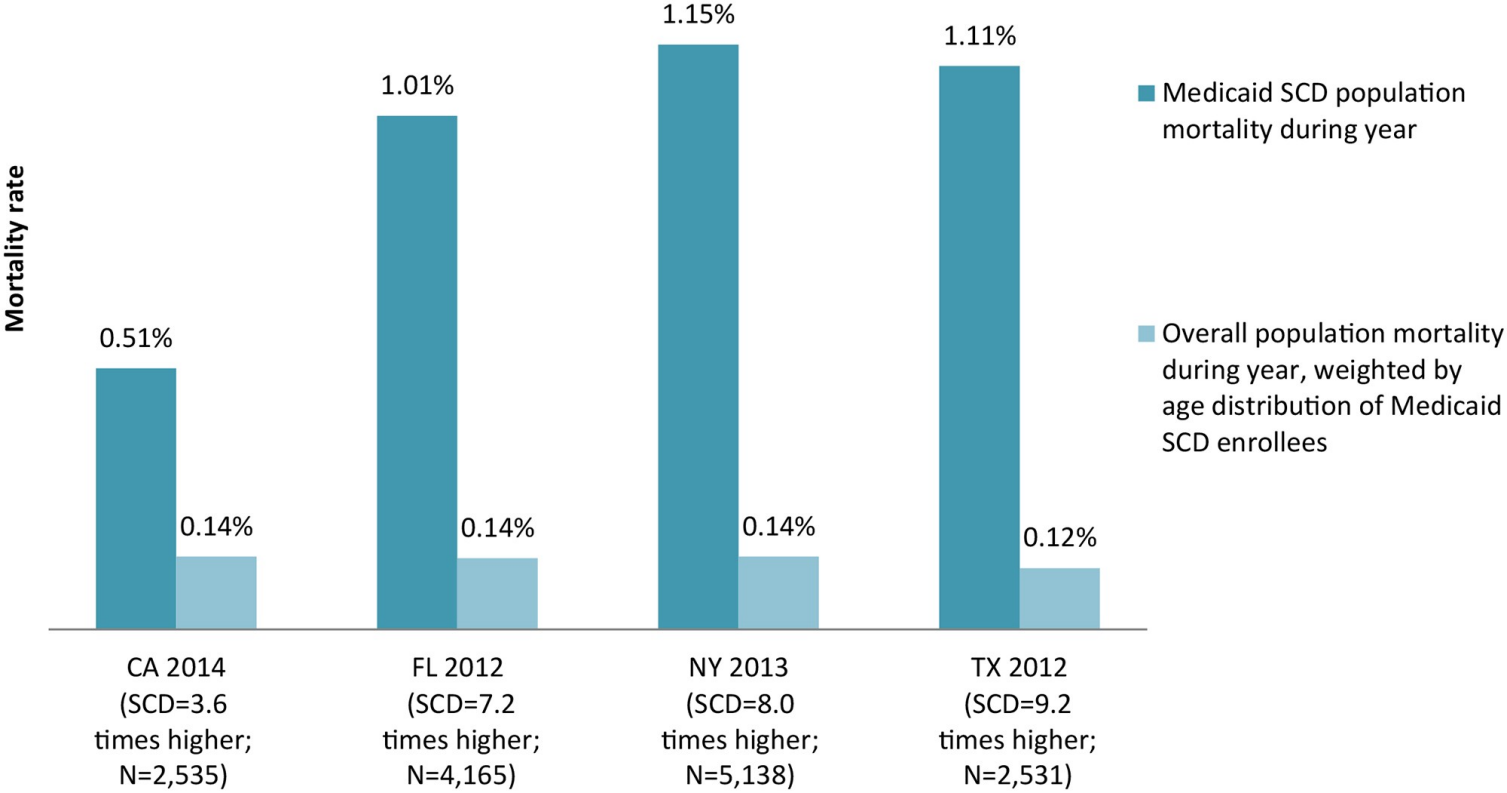
Mortality rate, Medicaid enrollees with SCD relative to age-adjusted population average. SCD is sickle cell disease.

Despite high utilization of available health care services, mortality for SCD enrollees can be more than nine times the age-adjusted population average, as observed for Texas (1.11 percent for those with SCD compared to a rate of 0.12 percent overall) (Fig 4).

## Discussion

**Fig 5:**
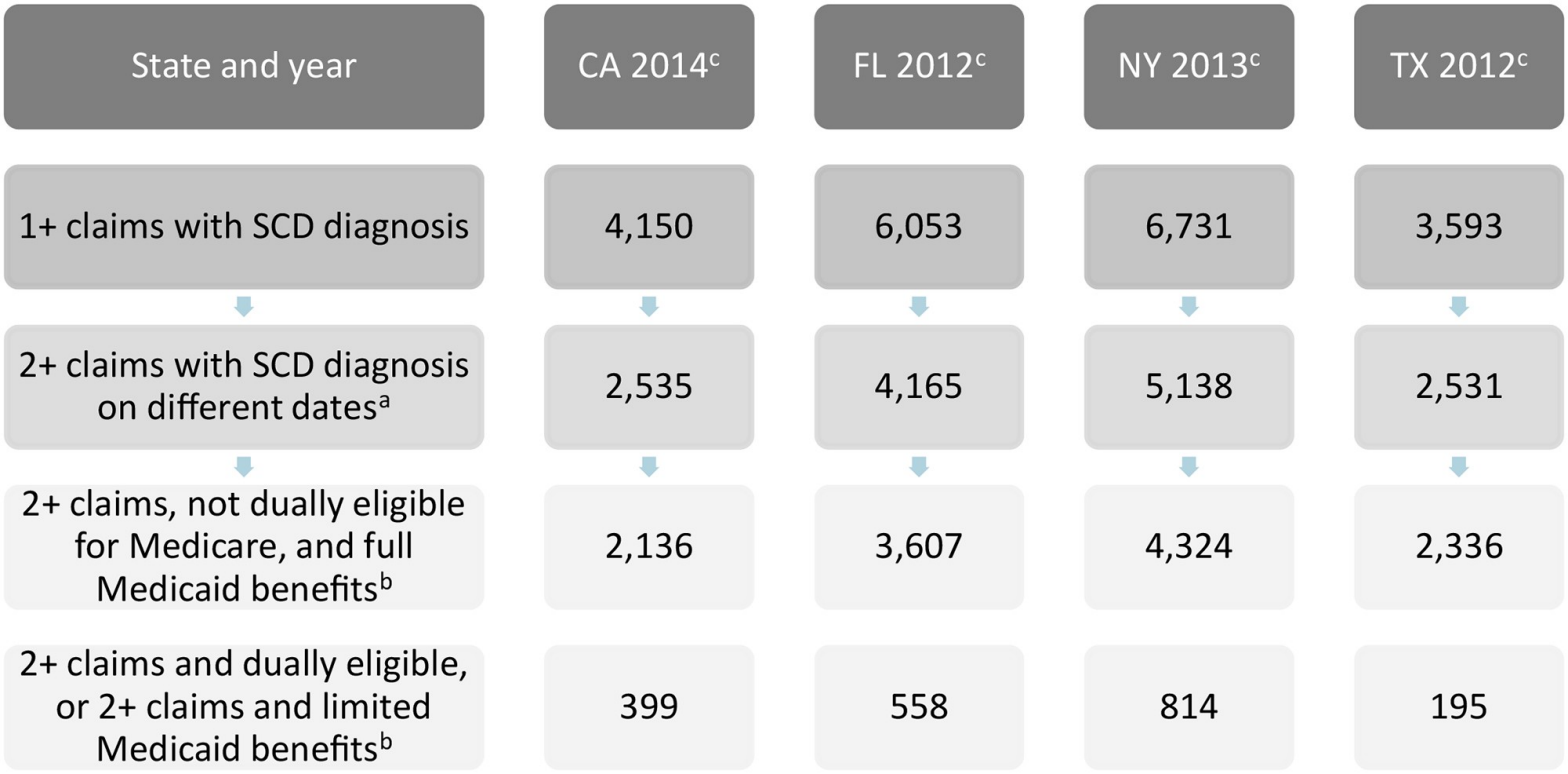
Medicaid SCD population size, by state and year. SCD is sickle cell disease.

The number of Medicaid enrollees with SCD is small relative to overall Medicaid program enrollment– 0.1 percent or less in each of the four states examined (Fig 5). This is consistent with a CMS finding that enrollees with SCD made up only 0.07 percent of total Medicaid enrollees nationally in 2012 [17].

**Fig 6:**
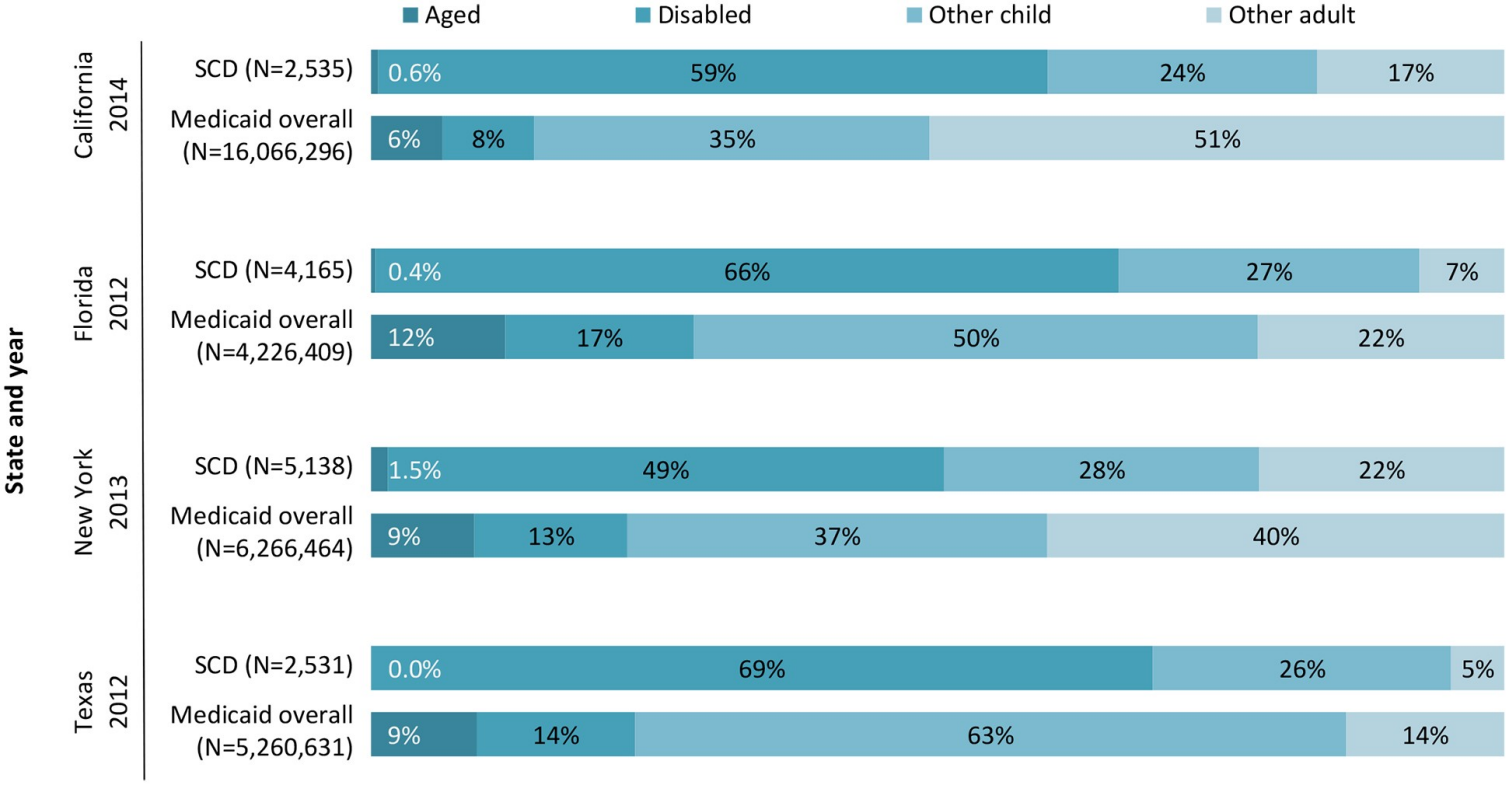
Distribution of Medicaid enrollment via disability-related versus other eligibility pathways, SCD relative to overall. SCD is sickle cell disease.

In California, SCD enrollees have rates of disability-related eligibility more than seven times the overall program average (Fig 6). The share of SCD enrollees in each eligibility pathway to Medicaid partly reflects state choices. For example, the “Other adult” category for Florida and Texas primarily reflects parents and pregnant women for whom the federal government mandates certain Medicaid coverage levels. For California and New York, this group also reflects state-optional coverage of childless adults on the basis of their low incomes alone, rather than their parental, pregnancy, or disability status (Fig 6).

Medicaid enrollees with SCD have substantially higher than average utilization of inpatient care, emergency department services, and opioids and other outpatient drugs. The proportion of Medicaid enrollees with an inpatient hospital stay is typically less than 10 percent overall [18, 19], versus nearly half or more for the SCD population (Fig 3). For the Medicaid population overall, about one-third have any emergency department visit per year [18, 19]. In comparison, 69 percent to 77 percent of the SCD population had an outpatient emergency department visit (i.e., excluding those that led to an inpatient stay) in the studied states (Fig 3). Similarly, the share of enrollees with an opioid outpatient prescription is about 15 percent for Medicaid overall [20], versus 50 percent or more for the SCD population (Fig 3).

Average and median Medicaid spending for enrollees with SCD can be more than five times that of all Medicaid enrollees in a state (e.g., averages of $24,800 versus $4,200 and medians of $9,500 versus $1,100 in Florida) (Fig 2). However, averages mask significant costs for those with an acute medical event, both within and across years. For example, 15 percent of SCD enrollees in California had at least four inpatient hospital stays during 2011 and spending per enrollee of more than $100,000, indicative of significant complications associated with their SCD and other health conditions. Following these individuals over time, more than 80 percent were enrolled in each of the years 2011–2014, and their average cumulative spending over those four years was $298,000. Further reinforcing the importance of managing significant health events for this population is the fact that SCD is among the top conditions observed in analyses of “super-utilizers” and potentially preventable hospital readmissions in Medicaid [21, 22].

In absolute terms, mortality among Medicaid enrollees with SCD is low, at about 1 percent annually. However, the figure is extraordinarily high given that nearly 80 percent of SCD enrollees in some states are under age 30, a group for whom mortality rates are typically less than 0.1 percent [16]. In addition, the 1 percent mortality rate for Medicaid enrollees with SCD is likely to be an underestimate due to incomplete reporting of deaths in the MAX data (Fig 4).

## Conclusion

This study’s results are aligned with prior analyses noting the high health care utilization and significant costs of the SCD population. A key contribution to the existing knowledge base is the finding that disability is a predominant pathway to Medicaid eligibility for those with SCD, which has wide-ranging implications. Because SCD is a debilitating condition that affects individuals’ day-to-day functioning and ability to work, its impacts on their health and well-being are lifelong. In turn, many individuals with SCD–particularly those who are impaired enough to qualify for disability benefits–will receive Medicaid coverage of their high health care needs on a long-term basis.

Another contribution of this study is its demonstration of state variation in the demographic, utilization, and spending characteristics of Medicaid enrollees with SCD. While this study focuses on four states that account for more than one-third of Medicaid enrollment, they may not be representative of the Medicaid SCD population at large and the full range of state experience nationwide, potentially limiting the generalizability of results.

Gene therapies currently under development to address the genetic cause of SCD may have durable clinical effects and could significantly change the current standard of care for people with SCD. As a result of the potential long-term benefits of these therapies, questions of appropriate coverage and reimbursement as well as expected outcomes will be at the forefront for Medicaid and other payers. Because Medicaid plays a substantial role in covering the cost of care for patients with SCD, state and federal officials will need to understand how the disease currently affects beneficiaries. Moreover, it will be important for these stakeholders to assess the impact of these gene therapies on patients’ quality of life and life span and, in turn, the potential of these therapies to reduce patients’ need for crisis-driven care and disability-related supports.
